# The Cognitive Profile of Mild Cognitive Impairment Due to Dementia With Lewy Bodies—An Updated Review

**DOI:** 10.3389/fnagi.2020.597579

**Published:** 2020-12-23

**Authors:** Mathilde Suhr Hemminghyth, Luiza Jadwiga Chwiszczuk, Arvid Rongve, Monica Haraldseid Breitve

**Affiliations:** ^1^Research Group for Age-Related Medicine, Haugesund Hospital, Haugesund, Norway; ^2^Department of Clinical Medicine (K1), University of Bergen, Bergen, Norway

**Keywords:** dementia with lewy bodies (DLB), mild cognitive impaiment, cognition, predementia, neuropsychological profiles, prodromal DLB

## Abstract

**Objective:** Dementia with Lewy Bodies (DLB) is the second most common type of neurodegenerative dementia. Yet, the domain-specific cognitive impairment of the mild cognitive impairment (MCI) phase of this disease (DLB-MCI) is still not been established. This article gives an updated review on the neuropsychological profile of DLB-MCI, building on the findings from a previous review.

**Methods:** We performed systematic review and searched five different electronic databases (Scopus, Cochrane, EMBASE, MEDLINE, and PsycINFO) in May 2020 based on a PICO scheme. Our search was then restricted to articles published in 2019 and 2020. Ending up with a total of 90 articles to be reviewed by abstract and/or full text.

**Results:** In total four papers were included, whereof only one met our full inclusion criteria. Despite a substantial heterogeneity, our findings indicate that DLB-MCI patients have a pattern of executive, visuospatial, and attentional deficits.

**Conclusion:** The findings indicate that the neuropsychological profile of DLB-MCI is characterized by executive, visuospatial, and attentional deficits. Furthermore, the shortage of studies clearly underlines the paucity of published research into DLB-MCI and emphasizes the need for well-controlled studies.

## Introduction

Dementia with Lewy bodies (DLB) comprises up to 24% of all neurodegenerative dementia cases (Hogan et al., 2016). It is thereby the second most prevalent type of neurodegenerative dementia, only exceeded by Alzheimer's Disease (AD), and the need for accurate and early diagnostic identification is thus of great social importance. DLB is characterized by dementia in combination with different core clinical features of rapid eye movement (REM) behavior disorder (RBD), parkinsonism, cognitive and alertness fluctuations, and visual hallucinations. In addition, there are a number of supportive clinical features, indicative biomarkers, and supportive biomarkers to be used when establishing a clinical diagnosis (McKeith et al., 2017).

Both Parkinson's disease (PD) with and without dementia (PDD) and DLB are thought to be on separate ends of a continuum of Lewy Body diseases (LBDs) (Aarsland et al., 2009). Thus, the two diagnoses share important pathophysiological and clinical features, including motor, neuropsychiatric, cognitive, and autonomic symptoms. Because of this extensive overlap, it has even been up for discussion whether PDD and DLB really are two distinct diagnoses (McKeith, 2009; Berg et al., 2014). Today DLB is diagnosed when dementia precedes or accompanies parkinsonism, whereas PDD is diagnosed when dementia occurs after PD onset. For research matters, the differentiation of DLB and PDD is defined by a 1-year criterion, where PDD is diagnosed when PD occurs a year or more before dementia (McKeith et al., 2017).

Moreover, studies also suggest that both neuropsychological deficits and core clinical features arises early in the disease progression (Sperling et al., 2011; Donaghy et al., 2015), and the phase preceding dementia has therefore been heavily investigated over the last years. This phase, called the mild cognitive impairment (MCI) stage, points to the intermediate stage between normal cognitive aging and dementia. Here objective cognitive decline is present, but not to the extent that it interferes with functional daily living. Research now suggests that the MCI stage due to the different dementia types have their unique cognitive profiles equal to their associated dementias. As of today, the MCI stage for both AD (AD-MCI) and PDD (PD-MCI) are defined (Albert et al., 2011; Litvan et al., 2012), but the proposed MCI stage for DLB (DLB-MCI) (Donaghy et al., 2018) is still lacking support from clinical studies. Indeed, research criteria for the diagnosis of prodromal DLB has only recently been published (McKeith et al., 2020), defining DLB-MCI as MCI with concurrent probable or possible DLB (McKeith et al., 2017, 2020).

Furthermore, the need for validation of the DLB-MCI criteria is especially pressing, as there is an overall trend in the field of dementia to advance the point of diagnosis to the MCI stage. The high rate of misdiagnosis, the symptomatic overlap between different diagnoses, and the fact that the different diagnoses calls for different treatment makes the validation even more exigent. Moreover, defining the cognitive profile of DLB-MCI might also be of important prognostic value, as neuropsychological tests is shown to be good predictors for conversion from MCI to AD (Belleville et al., 2017).

A recent review indicates that the cognitive profile of DLB-MCI is characterized by executive dysfunction, slow processing speed, in addition to visuospatial and working memory deficits (Ciafone et al., 2020). It thus resembles the cognitive profile for the PD-MCI. However, the authors emphasized the paucity of research into DLB-MCI and therefore the need for more studies. Given both the time passed since Donaghy et al. (2018) proposed a DLB-MCI and the importance of the area, one might expect that more research is now available.

## Methods

This is an updated and systematic review of the evidence base underlying the domain-specific cognitive impairment associated with DLB-MCI, building on a recently published review based on a search performed in January 2019 (Ciafone et al., 2020). This systematic review conforms to the PRISMA reporting guidelines.

### Search Strategy

Building on a PICO scheme (Supplementary Material), we searched Scopus, Cochrane, EMBASE, MEDLINE, and PsycINFO databases in May 2020 (2020-05-14 and 2020-05-22). Free text words were used in all databases: “Lewy” or “DLB” with “dementia” in combination with “prodromal^*^” and “cognit^*^ dysfunction/decline/impair^*^/defect^*^/deficit^*^,” MCI and/or SCD [subjective cognitive decline]. In addition to text words, medical subject headings from thesaurus were used when possible, thus in Medline and Cochrane Central (Lewy Body disease, prodromal symptoms, cognitive dysfunction), EMBASE (prodromal symptoms, diffuse Lewy body disease, dementia assessment, cognitive defect), and PsycINFO (dementia with Lewy bodies, prodrome, cognitive impairment). The search was restricted to title, abstract, and keyword. The search was performed in collaboration with a University librarian.

### Inclusion and Exclusion Criteria

We included studies that measured domain specific cognitive functioning in DLB-MCI. Both studies that reported scores on isolated neuropsychological tests as well as those including domain specific composite scores were accepted. On the other hand, our exclusion criteria were n < 10, lack of healthy control (HC) subjects, unclear diagnostic and MCI definitions, and use of global cognitive composite scores. That is, studies who only reported cognition in terms of global Mini-Mental Status Examination (MMSE) or Montreal Cognitive Assessment (MoCA) (Nasreddine et al., 2005) scores were excluded, as opposed to those who for example reported domain-specific MoCA subscores. Furthermore, studies including only one case of DLB-MCI in addition to other MCI groups were considered case studies, and thus removed.

## Results

**Figure 1:**
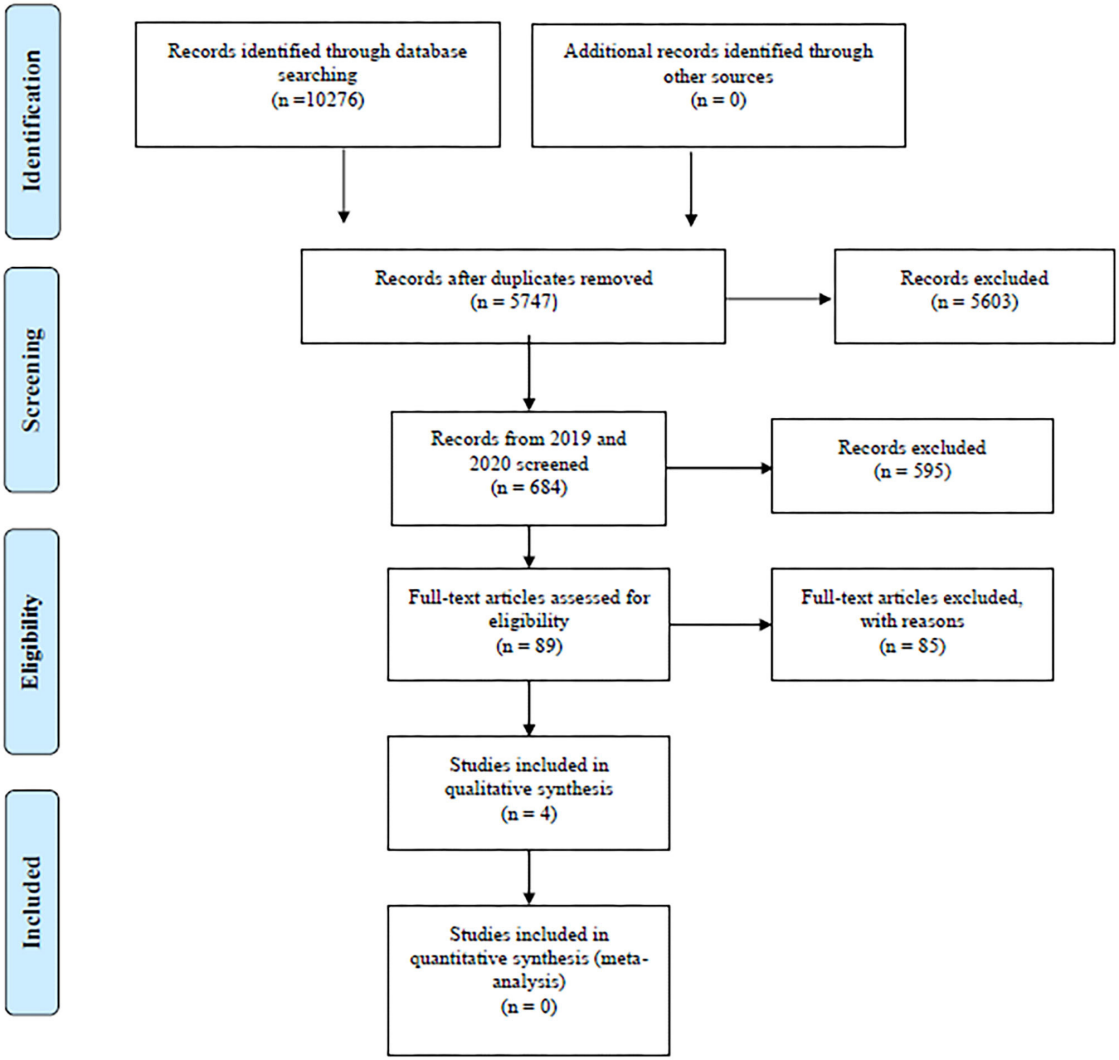
PRISMA flow diagram. Overview of search and extraction process. From: Moher et al. (2009). For more information, visit www.prisma-statement.org.

The search yielded a total of 10,276 references: 6,300 after removing duplicates using EndNote and 5,747 after manually removing remaining duplicates. These references were further restricted to those published in 2019 and 2020, resulting in a total of 684 references subjected for title screening. After a gross title screen, removing animal studies, articles with a flagrantly irrelevant topic or articles that were not published in English journals, 89 references remained to be reviewed by abstract. See Figure 1.

The abstract review typically removed case studies, and articles that did not target DLB did not include cognitive measures or only included global cognitive composite scores (such as MMSE or MoCA total score). The main reasons for removal after full text revision were studies that did not distinguish between prodromal symptoms and symptoms of (mild) dementia, unclear neuropsychological data, and lack of control groups. Reviews, meta-analyses, abstracts, posters, discussion papers, and commentaries were also screened out during the process. At both the point of abstract review and full text review, ambiguous cases were discussed between the main authors (MSH, MHB) until consensus was achieved. We extracted baseline data in longitudinal studies, unless otherwise stated.

Since only one study met the full inclusion criteria (Massa et al., 2019), we *post hoc* chose to include studies that did not have HCs and had unclear diagnostic criteria. Doing this we ended up with a total of four articles for this review (Massa et al., 2019; Unger et al., 2019; van de Beek et al., 2020; Yoo et al., 2020). See Table 1.

**Table 1:** Overview of the included studies.

**References**	**Country**	**Study type (time of inclusion)**	**Study aim**	**Patient group (n)**	**Control group (n)**	**MCI criteria**	**Diagnostic criteria**	**Neuropsychological tests**	**Findings: impaired functions in the patient group**
Massa et al., 2019	Italy	Longitudinal follow-up study (2004–2017)	To characterize the neuroimaging and neuropsychological characteristics of LBD-MCI	MCI-P (13).	HC (18) PD-MOT (11)	Impairment on ≥ two NP tests, preserved ADL/IADL, CDR = 0.5	PD as defined by Gelb et al. (1999)	MMSE, RAVLT, Babcock story recall Test, Corsi span, Digit span, TMT A-B, Stroop, Digit symbol test, figure copying. Clock drawing test, categorical, and phonological verbal fluency tests	Executive function, processing speed/ attention, visuospatial ability, memory
van de Beek et al. (2020)	The Netherlands	Retrospective cohort study (N/A)	To investigate the clinical characteristics and the predictors for dementia onset in prodromal DLB and AD.	MCI-LB (73)	MCI-AD (124)	Cognitive complaint, preserved ADL, MMSE ≥25, z ≤ −2 in one NP domain	Probable DLB (McKeith et al., 2017)	MMSE, Visual Association Test, verbal learning test, TMT A-B, Stroop 1-3, Digit span, Letter fluency, Frontal Assessment Battery, VOSP number-location, dot counting, fragmented letters, Visual Association Naming Test	Executive and visuospatial function
Unger et al. (2019)	USA	Retrospective cohort study (2016–2018)	To characterize the diagnostic value of CVI testing, by comparing the CVI of patients with DLB, AD and pro-LBD.	pro-LBD (25)	DLB (62)	N/A	RBD and/or parkinsonism. No prior diagnosis PD or Parkinson clinical syndromes	MoCA	Visuospatial/executive, attention, memory deterioration
Yoo et al., 2020	South Korea	Cross-sectional study (2015–2018)	To evaluate the striatal dopaminergic depletion and cerebral beta-amyloid deposition, and their association to cognitive function in LBD patients.	LBD-MCI (18)	HC (15)	N/A	Probable DLB (McKeith et al., 2017) and level-II PD-MCI (Litvan et al., 2012)	MMSE, Digit span, BNT, RCFT, SVLT, semantic and phonemic COWAT, Stroop	Attention, memory, executive function

*MCI-P, patients where MCI was preceding Parkinson's disease; HC, healthy controls; PD-MOT, cognitively normal PD patients; NP, neuropsychological; DLB, dementia with lewy bodies; MCI-LB, MCI due to lewy body dementia; MCI-AD, dementia due to Alzheimer's disease; ADL, activities of daily living; IADL, instrumental ADL; Pro-LBD, prodromal lewy body disease; RBD, rapid eye movement (REM) sleep behavior disorder; CVI, color-vision impairment; MMSE, mini mental state evaluation; RAVLT, rey auditory verbal learning test; TMT, trail making test; VOSP, visual object and space perception battery; MoCA, montreal cognitive assessment; BNT, Boston naming test; RCFT, rey complex figure test; SVLT, Seoul verbal learning test; COWAT, controlled oral word association*.

### Synthetisized Findings

The only study that met the full inclusion criteria was a longitudinal study by Massa et al. (2019). The DLB-MCI patients were diagnosed with MCI prior to PD, with a mean time of 2.9 ± 1.9 years (range 0.5–6.1). These patients were then matched and compared to both HCs and cognitively normal PD patients. However, they only used cognitively normal PD patients instead of HCs in the analysis of cognitive data. They report heterogeneous findings at the baseline, with the cognitive profiles of DLB-MCI both being amnestic, non-amnestic and single- or multidomain. They did nevertheless find The Clock Drawing test and the TMT-B to be the most impaired cognitive tests in DLB-MCI. Compared to cognitively normal PD patients, the DLB-MCI patients had significantly reduced scores on tests measuring executive, attentional, visuospatial, and memory function. They found one test of visuospatial working memory and one of semantic verbal fluency to be able to fully separate those who develop LBD dementia from those who do not. The authors concluded that a worse visuospatial working memory and semantic verbal fluency is among factors putting a patient at higher risk of LBD dementia development.

Combining this with the neuroimaging findings, the authors conclude that patients with cognitive complaints and PD is best characterized as prodromal LBD (Donaghy and McKeith, 2014; McKeith et al., 2016) at first presentation, due to the extensive pathophysiological overlap.

The last three included studies did not, for various reasons, meet our full inclusion criteria. van de Beek et al. (2020) did not include HCs, Unger et al. (2019) lacked both HCs and had an unclear MCI definition, and lastly Yoo et al. (2020) both had an unclear MCI definition and a combined PD/DLB-MCI group.

Despite this, summed findings do point to DLB-MCI patients having poorer executive and visuospatial function compared to AD-MCI patients (van de Beek et al., 2020) and poorer executive and attentional functioning compared to HCs (Yoo et al., 2020). Moreover, a deterioration in attentional (Unger et al., 2019; van de Beek et al., 2020) and visuospatial/executive function (Unger et al., 2019) has been reported for DLB-MCI patients converting to dementia. Interestingly, van de Beek et al. (2020) also found that a poorer attentional function at first visit was associated with faster progression to dementia. The authors then concluded that this probably is an effect of these patients being closer to dementia, as DLB is characterized with more prominent attentional dysfunction early on in the disease course. Hence, the results point to a pattern of executive, visuospatial, and attentional deficits for persons living with DLB-MCI. Even so, the few existing studies provide rather heterogeneous findings, which is in line with Ciafone et al. (2020)'s findings.

When it comes to memory, the results are somewhat more mixed. While some of the studies find memory to be spared compared to AD-MCI (van de Beek et al., 2020), others find memory to be affected in DLB-MCI compared to HCs (Yoo et al., 2020). Lastly, both van de Beek et al. (2020) and Unger et al. (2019) report memory deterioration to be associated with conversion to dementia.

## Discussion

Despite being already 2 years since Donaghy et al. (2018) proposed criteria for DLB-MCI, only one study met the full inclusion criteria for this review (Massa et al., 2019). Further, only one study focused primarily on cognition (van de Beek et al., 2020), while the others had either color vision impairments or neuroimaging as their main focus. This shortage clearly underlines the paucity of research into DLB-MCI and emphasizes the need for well-controlled studies. Thus, the situation seems to be quite similar to that of January 2019 (Ciafone et al., 2020), and the cognition of DLB-MCI is still a scarce field of research. However, there are well-designed ongoing studies, that is, the Dementia Disease Initiation (DDI)-study (Fladby et al., 2017), that focuses on prodromal dementia symptoms, including prodromal DLB symptoms.

Because the main part of the included studies did not meet our full inclusion criteria, it is timely to question their utility for the scope of this review. Either way, the findings do however push toward a neuropsychological profile characterized by visuospatial, executive, and attentional deficits. In fact, this is in line with what we also found in mild DLB (Brønnick et al., 2016).

Furthermore, Massa et al. (2019) also found a worse visuospatial working memory and semantic verbal fluency to be a risk factor for LBD dementia development. One might question the specificity of this finding because it is in line with what is also found to be the case for conversion from MCI to AD (Belleville et al., 2017). Moreover, the results are inconclusive when it comes to the memory function of DLB-MCI patients. One might theorize that the large heterogeneity is due to some patients having a mixed DLB-AD pathology. However, caregivers to patients with DLB report memory impairment to be the most common symptom for both DLB and AD (Auning et al., 2011).

In addition, the substantial heterogeneity in the reported findings may firstly be due to the considerable variation in applied methods and definitions. One of the included studies used domain specific screening tools (Unger et al., 2019), while the others used more exhaustive neuropsychological tests. Further, some used composite scores (van de Beek et al., 2020; Yoo et al., 2020), while other analyzed isolated test scores (Massa et al., 2019; Unger et al., 2019). Moreover, some of the studies used standardized scores in the analyses (van de Beek et al., 2020; Yoo et al., 2020), while others used raw scores (Massa et al., 2019; Unger et al., 2019). This is somewhat problematic, due to age, sex, and educational differences between the subjects, as well as substantial differences in the normative basis for the different tests.

Second, we do not know whether all of the patients diagnosed as prodromal DLB did actually develop DLB. Only two of the included studies were longitudinal (Massa et al., 2019; van de Beek et al., 2020), and in these studies just about half of the subjects diagnosed as DLB-MCI actually developed dementia. In addition, in van de Beek et al. (2020), patients who received a probable DLB diagnosis at some point during follow-up were retrospectively defined as DLB-MCI (44%). Hence, these subjects probably did not reach the same criteria for a clinical diagnosis DLB-MCI at first presentation as the other ones, and grouping these together is thus problematic with respect to operationalization. The ideal situation would be to have a group of patients clinically diagnosed with DLB-MCI who later on received a definite DLB diagnosis.

Third, the heterogeneity of the findings may also be a natural cause of the varying terms and diagnostic criteria used in the different studies. Indeed, only van de Beek et al. (2020) stated a clear MCI definition together with McKeith et al. (2017) criteria for probable DLB. The other studies chunked PDD and DLB together to an LBD-MCI group, or had a restricted focus on parkinsonism or RBD, not taking the other DLB core criteria into account. For example, Massa et al. (2019) did not use the 1-year rule as is stated for research purposes to differentiate between PDD and DLB. Furthermore, Yoo et al. (2020) also chunked PDD and DLB together in an LBD-MCI group, of which only four of them were diagnosed as DLB-MCI, as opposed to 14 as PD-MCI. This bias clearly affects the utility of this study as a DLB-MCI study; in fact, it may be more representable as a PD-MCI study.

Future studies must therefore apply the newly published consensus criteria (McKeith et al., 2020) to make comparison between studies possible. Here MCI is defined as a cognitive concern proposed by the patient, an informant, or a clinician, in addition to objective impairment in one or more cognitive domains and preserved ADL. In order to receive a diagnosis of DLB-MCI, the patients must also meet the criteria for probable or possible DLB (McKeith et al., 2017, 2020). In the time ahead, we accordingly expect a substantial amount of studies using these new criteria. Despite the budding amount of research suggesting visuospatial, attentional, and executive deficits in DLB-MCI, a sound conclusion regarding the cognitive profile of DLB-MCI awaits a future body of research using the McKeith et al. (2020) criteria. Additionally, studies with higher focus on new potential biomarkers and a larger number of included subjects is important to further subgroup this heterogeneous group and to get more robust and representative findings.

Lastly, some of the included studies proposed that a DLB-MCI diagnosis might not be fruitful, due to a both substantial symptomatic heterogeneity and overlap (Massa et al., 2019; van de Beek et al., 2020). At this point, however, it seems like a rather premature proposition due to the lack of well-controlled studies. As mentioned, Massa et al. (2019) suggests a prodromal LBD diagnosis for patients with both PD and cognitive complaints, due to their finding of a substantial pathophysiological overlap between PD-MCI and DLB-MCI patients. This may, however, be a spurious effect because they did not adhere to the 1-year rule and chunked prodromal PDD and DLB patients together in their prodromal LBD group. Hence, a pathophysiological overlap is as expected. Nonetheless, this discourse points to the necessity of conducting proper neuropsychological assessments of all patients with PD.

### Study Limitations and Strengths

The strength of this study is the thorough and systematic search in which it is based on, thus making it unlikely that we missed relevant studies. Our study does, however, have some limitations, with the most obvious being the few studies included. All of the studies also had few study subjects included. One can therefore not use the findings from this study to validate the neuropsychological profile of DLB-MCI. Another minor limitation is that we are building on a recently published review without knowing the exact search strategy of this study. Moreover, our searches were not performed in the exact same databases as Ciafone et al. (2020).

## Conclusion

As Ciafone et al. (2020), our findings indicate that DLB-MCI patients have a pattern of executive, visuospatial, and attentional deficits. Hence, it resembles that of DLB. However, there is a clear lack of well-controlled studies with a sizable number of study subjects focusing on cognition in DLB-MCI. One major reason for this might be the challenge of identifying these patients due to both heterogeneous symptom presentation in the prodromal stage and the lack of clear definition. With recently published research criteria for DLB-MCI (McKeith et al., 2020), it might be easier to identify these patients, and a wealth of studies is therefore expected to come in the near future.

## Data Availability Statement

The original contributions presented in the study are included in the article/Supplementary Material, further inquiries can be directed to the corresponding author.

## Author Contributions

MH conceived the research question, which was then discussed with all the authors. MH performed the searches and drafted the manuscript. MB was the main supervisor. At inclusion ambiguous cases were discussed between MH and MB. LC and AR contributed to the hypothesis and revised the manuscript. At the end all authors read and approved the final version of the manuscript.

## Conflict of Interest

The authors declare that the research was conducted in the absence of any commercial or financial relationships that could be construed as a potential conflict of interest.
