# Supplementary material for: The Cognitive Profile of Mild Cognitive Impairment Due to Dementia With Lewy Bodies—An Updated Review
Source: Front Aging Neurosci. 2020 Dec 23;12:597579. doi: 10.3389/fnagi.2020.597579 (PMC7785712; doi:10.3389/fnagi.2020.597579)
Supplement: Supplementary file 1 [file Data_Sheet_1.pdf]

Supplementary Material

**P: Population/patient/problem**	Patients diagnosed with mild cognitive impairment (MCI) as a precursor for DLB (DLB-MCI)
**I: Intervention**	Assessment of neuropsychological symptoms; what characterizes the cognitive decline of DLB-MCI
**C: Comparison**	Healthy controls
**O: Outcome**	The cognitive profile of DLB-MCI; validating the DLB-MCI as a unique diagnosis
**Study types:**	Cohort-studies, case-control studies, cross-sectional studies, longitudinal studies
